# Melatonin and Glycine Reduce Uterus Ischemia/Reperfusion Injury in a Rat Model of Warm Ischemia

**DOI:** 10.3390/ijms22168373

**Published:** 2021-08-04

**Authors:** Viktorija Zitkute, Mindaugas Kvietkauskas, Vygante Maskoliunaite, Bettina Leber, Diana Ramasauskaite, Kestutis Strupas, Philipp Stiegler, Peter Schemmer

**Affiliations:** 1 General, Visceral and Transplant Surgery, Department of Surgery, Medical University of Graz, Auenbruggerplatz 2, 8036 Graz, Austria; viktorijazitkute@gmail.com (V.Z.); min.kvietkauskas@gmail.com (M.K.); philipp.stiegler@medunigraz.at (P.S.); peter.schemmer@medunigraz.at (P.S.); 2 Faculty of Medicine, Vilnius University, M. K. Ciurlionio 21, 03101 Vilnius, Lithuania; vygantem@gmail.com (V.M.); diana.ramasauskaite@santa.lt (D.R.); kestutis.strupas@santa.lt (K.S.); 3 National Center of Pathology, Affiliate of Vilnius University Hospital Santaros Klinikos, P. Baublio 5, 08406 Vilnius, Lithuania

**Keywords:** melatonin, glycine, ischemia and reperfusion injury, uterus transplantation

## Abstract

Ischemia/reperfusion injury (IRI) remains a significant problem to be solved in uterus transplantation (UTx). Melatonin and glycine have been shown to possess direct cytoprotective activities, mainly due to their antioxidative and anti-inflammatory properties. The aim of this study was to investigate the protective effects of melatonin and glycine and their combination on IRI in a rat model of warm ischemia. In this study, *Sprague-Dawley* rats were assigned to eight groups, including sham and IRI (*n* = 80). Melatonin and glycine alone or their combination were administered prior to 1 h of uterus ischemia followed by 1 h of reperfusion. Melatonin (50 mg/kg) was administered via gavage 2 h before IRI and glycine in an enriched diet for 5 days prior to intervention. Uterus IRI was estimated by histology, including immunohistochemistry, and biochemical tissue analyses. Histology revealed that uterus IRI was significantly attenuated by pretreatment with melatonin (*p* = 0.019) and glycine (*p* = 0.044) alone as well as their combination (*p* = 0.003). Uterus IRI led to increased myeloperoxidase expression, which was significantly reduced by melatonin (*p* = 0.004), glycine (*p* < 0.001) or their combination (*p* < 0.001). The decline in superoxide dismutase activity was significantly reduced in the melatonin (*p* = 0.027), glycine (*p* = 0.038) and combined treatment groups (*p* = 0.015) when compared to the IRI control group. In conclusion, melatonin, glycine and their combination significantly reduced oxidative stress-induced cell damage after IRI in a small animal warm ischemia model, and, therefore, clinical studies are required to evaluate the protective effects of these well-characterized substances in uterus IRI.

## 1. Introduction

Successful uterus transplantation (UTx) has been shown to be the best treatment option for patients with absolute uterus factor infertility (AUFI) [1]. AUFI specifies women who are unable to become pregnant or maintain pregnancy because of the absence of a uterus (due to Mayer-Rokitansky-Küster-Hauser syndrome, or hysterectomy due to uterine benign/malignant tumors or postpartum bleeding), or the presence of a uterus that is anatomically dysfunctional (Asherman syndrome, septate, bicornuate, arcuate, hypoplastic or myomic uterus) [2,3,4].

In 2014, Brännström et al. reported the first human live birth following UTx, arousing interest in research on UTx as AUFI treatment and resulting in at least 20 clinical trials and even more animal experiments currently taking place across the world [5]. The need for UTx is growing in addition to the increasing number of successful clinical trials worldwide. To date, about 100 human UTx have been performed with around 20 reported successful live births [2]. 

Unlike other solid organ transplantation (Tx), UTx is the only ephemeral type of Tx, where the transplant is not intended for life-long use but for a limited time in the recipient (until delivery), and this restricted duration greatly reduces the risk of the well-known long-term immunosuppressive-related side effects [6,7]. Although UTx is still at the stage of animal experiments and clinical trials, its necessity is undeniable, and it is only a matter of time before this procedure becomes routine worldwide. 

Transplant success is limited by ischemia/reperfusion injury (IRI), and researchers are still struggling to answer key questions about uterus tolerance to IRI [4]. During ischemia, severe imbalance of metabolic supply and demand occurs, subsequently causing tissue hypoxia [8,9]. Moreover, restoration of blood flow and reoxygenation itself paradoxically further enhance the activation of innate and adaptive immune responses and cell death programs (reperfusion injury) [10]. These processes increase the likelihood of short- and long-term complications, such as delayed graft function and acute or chronic rejection [11]. Different strategies to reduce IRI, such as optimization of organ perfusion and storing conditions, or the development of drugs targeting IRI, are under investigation [12,13].

Melatonin is a hormone produced by various tissues in the body, although the major source is the pineal gland in the brain [14,15]. It is naturally produced from the amino acid tryptophan and comprises biological activities such as immunoregulatory, antioxidative and anti-inflammatory effects, as well as the ability to stabilize cell membranes; furthermore, its metabolites are able to reduce free radicals [14,16,17,18]. Melatonin receptors are expressed in a variety of cell types in rodents or primates as well as in the human female reproductive system, including the ovary, uterus, breast and placenta [14,19]. The potentially beneficial effects of melatonin have been shown in various animal models of IRI [12,20,21,22].

Glycine, the simplest amino acid, is involved in the synthesis of a variety of biomolecules and metabolisms, and is also an inhibitory neurotransmitter and an inhibitor of the activation of immune cells [23]. It is able to attenuate hypoxic cell injury by direct cytoprotection in isolated cells, organ perfusion and in vivo models targeting different organs, including the heart, liver, kidney and skeletal muscle [23,24,25,26,27,28].

This study was thus designed to evaluate the protective effects of melatonin and glycine, as separate supplements or in combination, on IRI in a rat model of warm ischemia.

## 2. Results

### 2.1. General Data

All animals were in good general health throughout the study period. One rat (1.25%) died after induction of anesthesia prior to surgery. The median body weight slightly increased from 303.9 (296.9; 322.1) g at the beginning of the study to 313.6 (304.9; 329.7) g at the end (*p* = 0.001). Five days of different diets (casein vs. glycine) did not affect body weight gain (3.04% (0.66; 6.84) vs. 2.7% (1.01; 4.42), *p* = 0.494), despite differences in median daily food intake (22.3 (21.4; 23.6) vs. 20.3 (19.3; 21.6) g, *p* < 0.001).

### 2.2. Glycine Concentration

**Figure 1:**
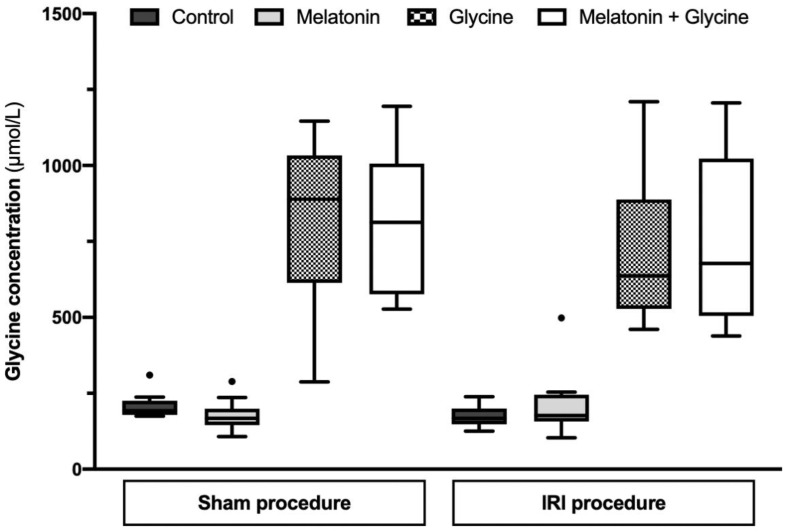
Glycine concentration in serum after 5 days of pretreatment in study groups. IRI: ischemia/reperfusion injury. Data presented as median and interquartile range (*n* = 10/group, except *n* = 9 in sham combined treatment group).

The median glycine concentration in serum, before switching to special diets (baseline), was 342.6 (303.3; 412.5) µmol/L. After 5 days of a glycine-enriched diet, median serum glycine concentration was 4.2-fold higher compared to the casein diet group (745.1 (558.7; 991.2) vs. 176.9 (155.4; 220.6) µmol/L, *p* < 0.001). The casein diet resulted in decreased glycine concentration compared to baseline values (*p* < 0.001), while the glycine-enriched diet was associated with significantly higher serum glycine levels (*p* < 0.001). The gavage with melatonin, 2 h before IRI or sham procedure, did not affect glycine levels at the end of the experiment (Figure 1). 

### 2.3. Histology

**Figure 2:**
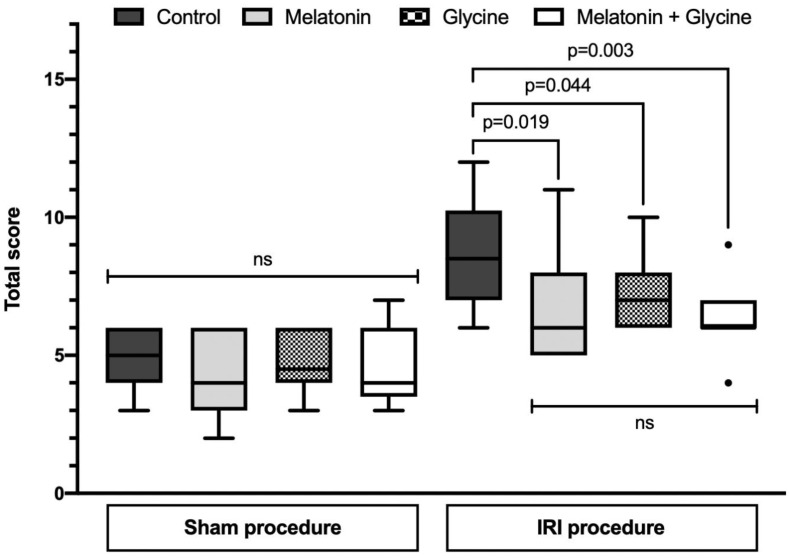
Total score of uterus histological evaluation for IRI. IRI: ischemia/reperfusion injury. Data presented as median and interquartile range (*n* = 10/group, except *n* = 9 in sham combined treatment group).

The total scores of uterus histological evaluation for IRI in all sham groups (control, melatonin, glycine and their combination) were similar (5 (4; 6) vs. 4 (3; 6) vs. 4.5 (4; 6) vs. 4 (3.5; 6), *p* = 0.719, respectively) (Figure 2). Uterus IRI procedure led to an increase in total scores, which were found to be 8.5 (7; 10.25) in control (*p* < 0.001), 6 (5; 8) in melatonin (*p* = 0.005), 7 (6; 8) in glycine (*p* < 0.001) and 6 (6; 7) in the combined treatment group (*p* = 0.022) compared to corresponding sham groups. The elevation in total score after IRI procedure was significantly attenuated by pretreatment with melatonin (*p* = 0.019) and glycine (*p* = 0.044) alone as well as with their combination (*p* = 0.003) compared to IRI control group.

When comparing the individual histological features of the scoring system between IRI groups, there was a significant reduction in tissue edema (*p* = 0.003) after pretreatment using the combined treatment. In addition, there was a tendency toward reduction in inflammatory cells (*p* = 0.074) and tissue edema (*p* = 0.068) after pretreatment with melatonin, while the same occurred with tissue edema (*p* = 0.074) and perimeter thickening (*p* = 0.068) after pretreatment with glycine, and with inflammatory cells (*p* = 0.068) after pretreatment of their combination (Table 1). Other histological features were similar in all IRI groups.

### 2.4. Tissue MPO Expression

**Figure 3:**
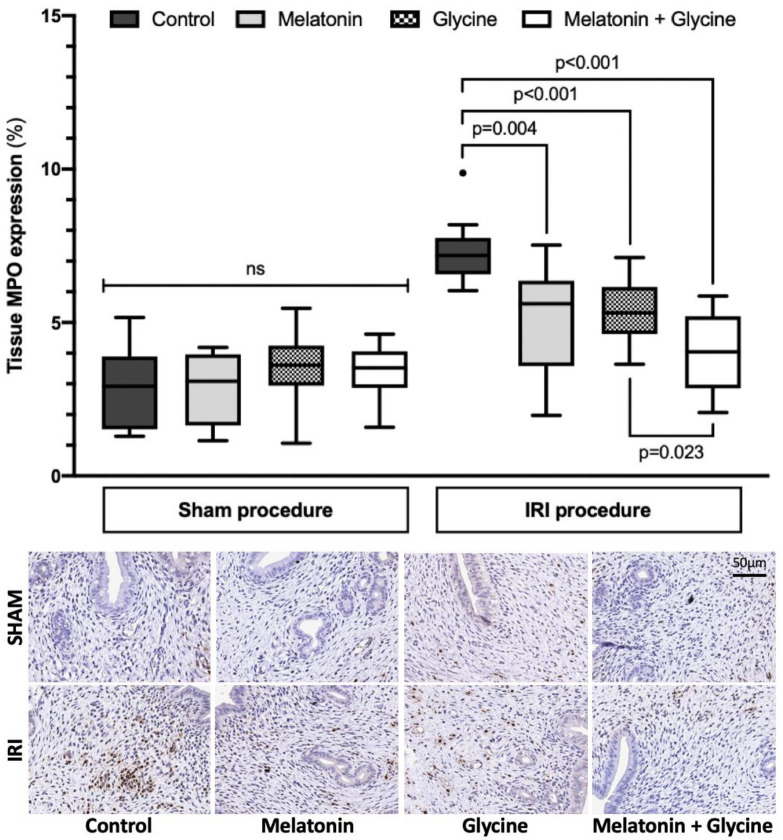
Myeloperoxidase expression in uterus tissue. IRI: ischemia/reperfusion injury. MPO: myeloperoxidase. Data presented as median and interquartile range (*n* = 10/group, except *n* = 9 in sham combined treatment group).

MPO levels in uterus tissue did not significantly vary between sham groups (Figure 3), with 2.92% (1.52; 3.89), 3.08% (1.64; 3.96), 3.61% (2.94; 4.25) and 3.52% (2.87; 4.06) in the sham control group, the sham melatonin group, the sham glycine group and the combined treatment group, respectively. Uterus IRI resulted in increased MPO levels (7.18% (6.57; 7.75) in the control group, 5.61% (3.58; 6.36) in the melatonin group, 5.31% (4.62; 6.16) in the glycine group and 4.04% (2.86; 5.2) in the combined treatment group). Pretreatment with melatonin (*p* = 0.004) and glycine (*p* < 0.001) alone or with their combination (*p* < 0.001) attenuated MPO increase following IRI compared to the IRI control group. While the combination treatment of glycine plus melatonin totally blocked the IRI effect of MPO expression (*p* = 0.224), all other IRI groups were different to their corresponding controls with *p* < 0.001, *p* = 0.005 and *p* = 0.002, for IRI, IRI plus melatonin and IRI plus glycine, respectively. 

### 2.5. Tissue SOD Activity

**Figure 4:**
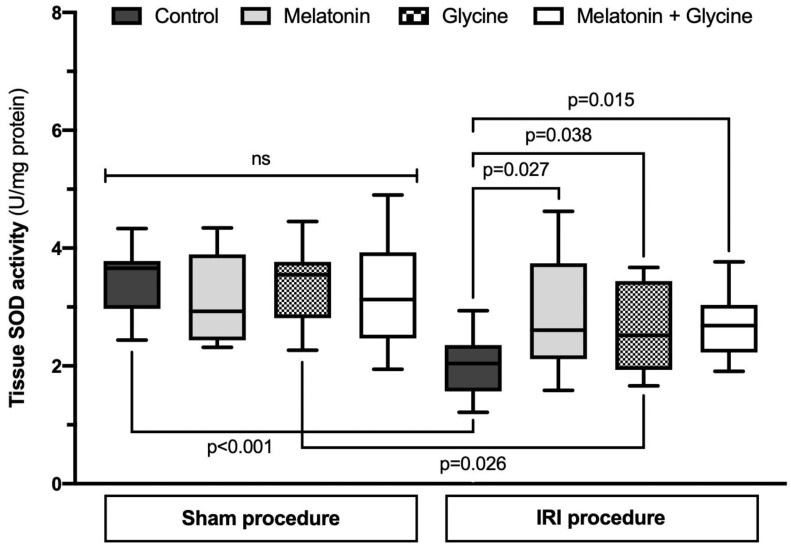
Superoxide dismutase activity in uterus tissue. IRI: ischemia/reperfusion injury. SOD: superoxide dismutase. Data presented as median and interquartile range (*n* = 10/group, except *n* = 9 in sham combined treatment group).

The SOD activity in uterus tissue samples was similar in all sham groups with 3.66 (2.97; 3.78) in control vs. 2.93 (2.44; 3.89) in melatonin vs. 3.55 (2.81; 3.77) in glycine vs. 3.13 (2.47; 3.93) U/mg protein in combined treatment group, *p* = 0.699) (Figure 4). IRI significantly decreases SOD activity. This decrease was attenuated by pretreatment with melatonin (2.61 (2.12; 3.74) U/mg protein, *p* = 0.027), glycine (2.52 (1.93; 3.44) U/mg protein, *p* = 0.038) and the combination of both supplements (2.69 (2.23; 3.04) U/mg protein, *p* = 0.015) as compared to the corresponding IRI control group (2.04 (1.57; 2.35) U/mg protein).

## 3. Discussion

In organ Tx IRI is a major challenge affecting clinical outcome. An imbalance in metabolic supply and demand within the ischemic organ results in profound tissue hypoxia and microvascular dysfunction; furthermore, subsequent reperfusion enhances the activation of innate and adaptive immune responses and cell death programs, culminating in acute or chronic organ rejections [10,29]. To date, only a limited number of studies investigating agents capable of minimizing IRI, particularly in UTx, are available [4]. The data of this study clearly demonstrate the protective effects of melatonin and glycine, both separately and combined, against experimental uterus IRI. This is the first implication of these non-toxic substances in the research of UTx.

The potential benefits of melatonin in other solid organ Tx have been described previously [12,16]. Melatonin is a potent free radical scavenger of reactive oxygen species (ROS) and, therefore, improves morphology, apoptosis, immunological reaction, and oxidative stress of grafts, among other processes. [16,30]. It has been proven to be a potentially useful therapeutic tool in reducing graft rejection [8]. In the last decades, its positive effects on the heart [31,32,33], bone [34], lung [35], pancreas [36,37], kidney [38,39,40] and liver [41,42,43] have been described in the Tx setting. Melatonin ameliorates IRI most likely through its antioxidative properties and inhibitory capacity of nuclear factor κB (NF-κB), IκB kinase (IKK) and c-Jun N-terminal kinase (JNK) in the mitogen-activated protein kinase (MAPK) pathway [40,44,45,46]. In addition, the capability of melatonin to enhance Akt activation in the setting of IRI has been documented previously [45,46,47]. All of these factors play an important role in inflammation and cell death. Our results demonstrated reduced histopathologic damage after uterus IRI. Moreover, the observed decline in SOD activity after 1 h of ischemia followed by 1 h of reperfusion was significantly attenuated by pretreatment with melatonin compared with control groups, leading to reduced oxidative stress. As a result, further polymorphonuclear infiltration and tissue activation, as documented by reduced MPO levels, significantly decreased with melatonin supplementation.

Numerous experimental and clinical studies have suggested the great potential of glycine in providing protection against IRI [24,25,48,49]. However, this protective ability does not apply to all organs and conditions. For the liver, heart and small intestine, several reports demonstrating organ protection have been published, while in the case of the kidney, it appears to be unlikely [23]. It seems that glycine attenuates IRI through similar pathways to melatonin: by enhancing Akt activation, while reducing the activation of extracellular signal-regulated kinase (ERK), JNK, and p38 in the MAPK signaling pathway [26,50,51]. Within this study, glycine mediated similar effects to those reported for melatonin. Glycine was able to preserve the activity of antioxidant pathways during uterus IRI, resulting in reduced oxidative stress and inflammatory cell activation as shown by MPO expression. Interestingly, the combination of melatonin and glycine did not result in significant additive effects as supposed, giving rise to new questions that may be answered by further investigations.

There were a number of limitations in our study. First, this model (1 h of ischemia and 1 h of reperfusion) did not reflect a real clinical situation. However, the time allowed for these processes was sufficient to trigger tissue-oxidative damage pathways and to investigate the effects of potentially beneficial agents. Second, the uteri exposed to IRI were not transplanted. However, as the effects of melatonin and glycine were being investigated in a model of uterus IRI for the first time, this study focused on answering preliminary questions. Within this study, melatonin and glycine revealed antioxidative and anti-inflammatory properties in uterus IRI, but further research is warranted. Novel therapeutic strategies are necessary, as minimizing IRI during Tx may potentially suppress the immune response against the allograft, leading to reduced need of systemic immunosuppression, thereby reducing additional risks [52], especially during pregnancy after UTx.

## 4. Materials and Methods

### 4.1. Animals

A total of 80 adult female (12-week-old; weight 270–360 g) *Sprague-Dawley* rats were obtained from Janvier Labs (Le Genest-Saint-Isle, France) and arrived at the research facility 7 days prior to intervention. All rats were kept under a controlled environment (22 ± 1 °C; 12 h/12 h light/dark cycle) and had access to fresh water and chow ad libitum. The study followed the guidelines for the handling and care of experimental animals issued by the Federation of European Laboratory Animal Science Associations (FELASA) and was approved by the Austrian Federal Ministry of Education, Science and Research (BMBWF-66.010/0197-V/3b/2018, 26 November 2018). 

### 4.2. Animal Groups and Experimental Design

**Figure 5:**
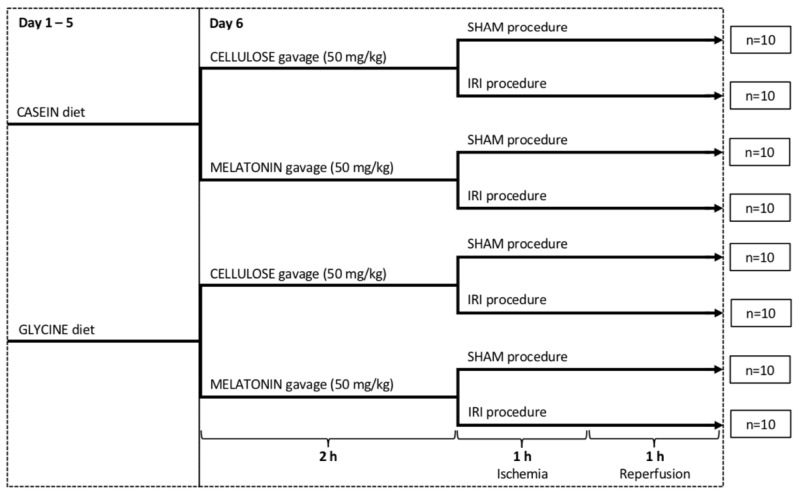
Study design. IRI: ischemia/reperfusion injury.

The rats (*n* = 80) were randomly assigned to either sham groups or experimental groups (*n* = 10/group) and subjected to a 7-day acclimatization period. Half of the animals in each group were then switched to a 5% glycine-enriched diet (containing 15% casein and 5% glycine for the glycine and combined treatment groups), while the other half received a control diet (containing 20% casein and 0% glycine for the control and melatonin groups), purchased from Altromin International (Lage, Germany), for 5 days. Two hours prior to IRI or sham procedure, the melatonin and combined treatment groups received 1.5 mL of milk (3.5% fat) containing 50 mg/kg melatonin (Sigma-Aldrich, St. Louis, MO, USA) via gavage, while the control and glycine groups received the same type of milk containing a corresponding amount of microcrystalline cellulose (placebo; from Sigma-Aldrich, St. Louis, MO, USA). The administration route and dose of both investigated substances (melatonin and glycine) were adopted based on previous experiments [27,28,40,44,53,54]. Water consumption, food intake and body weight were recorded regularly (for details, see Figure 5).

### 4.3. IRI and Sham Procedure

**Figure 6:**
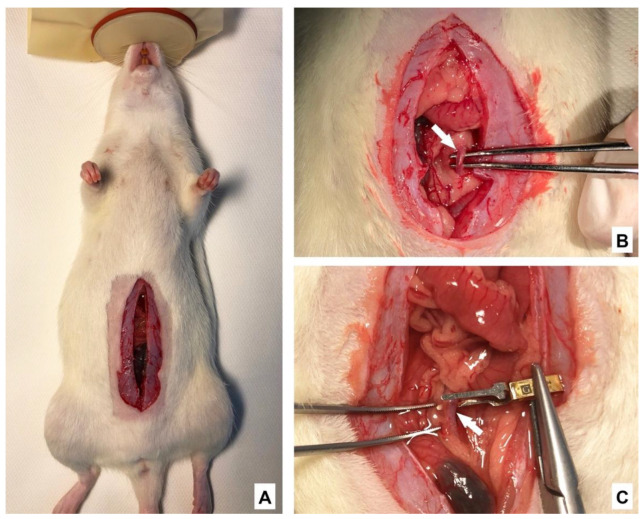
Surgical procedure. (**A**): laparotomy. (**B**): mobilization of the abdominal aorta (white arrow) about 0.5 cm above the bifurcation. (**C**): micro-bulldog clamp to be placed on the aorta (white arrow).

Anesthesia was performed using 2%, 2 L/min isoflurane inhalation and intramuscular injection of fentanyl (5 µg/kg), midazolam (2 mg/kg) and medetomidine (0.15 mg/kg). Animals were placed in a supine position on an automatically regulated heating pad to maintain normothermia during intervention. After shaving and disinfecting the surgical area, a horizontal laparotomy (measuring about 3 cm) was performed (Figure 6A). Ischemia was induced by clamping the distal abdominal aorta about 0.5 cm above the bifurcation with a micro-bulldog clamp (clamping pressure 20–25 g; GEISTER Medizintechnik, Tuttlingen, Germany; Figure 6B,C). Subsequently both ovarian arteries, including the surrounding fatty tissue, were temporarily ligated with Vicryl 3-0 sutures. The abdomen was closed and the animal was returned to a prone position. Warm ischemia was maintained for 1 h followed by relaparotomy restoration of the uterus blood flow by removing the micro-bulldog clamp and sutures from the ovarian arteries (see Figure S1 in Supplementary Materials). Reperfusion was maintained with closed abdomen in a prone position for 1 h. The duration of warm ischemia and reperfusion was based on previous preclinical studies and clinical case series [2,55,56,57]. At the end of the reperfusion period, animals were euthanized by terminal blood collection from the vena cava inferior. The right uterus horn was fixed in 4% formalin and prepared for histology and immunohistochemistry (IHC), while the left uterus horn was frozen in liquid nitrogen and stored at −80 °C for further biochemical analysis.

The sham procedure was performed in exactly the same manner, omitting vessel occlusion.

### 4.4. Histology

Paraffin-embedded samples were cut in 2 μm thick sections, stained with hematoxylin and eosin (H&E) [58], and subsequently examined by an experienced pathologist under a light microscope in a blinded manner. A modified semi-quantitative morphological scoring system was used for histological evaluation (Table 2; for details, see Supplementary Information) [59,60].

### 4.5. IHC Staining

The expression of myeloperoxidase (MPO), the oxidative stress marker and the indicator of neutrophils accumulation [61] was assessed by IHC staining with a rabbit polyclonal antibody to human MPO (Dako, Via Real, Carpinteria, CA, USA; dilution 1:800) in combination with the UltraVision LP Detection System HRP Polymer (Thermo Fisher Scientific, Waltham, MA, USA) and DAB chromogen (Dako, Via Real, Carpinteria, CA, USA). Rat spleen tissue was used as positive control, while primary antibody was omitted as negative control. The slides were scanned using the QuPath software version 0.2.0-m5 (Belfast, Northern Ireland) [13,62] and analyzed by a blinded examiner. Results are given as the ratio of MPO-positive cells to the total number of cells (percent MPO-positive cells).

### 4.6. Biochemistry

Frozen tissue samples were homogenized in 2 mL MagNA Lyser Green Beads tubes (Roche Diagnostics, Mannheim, Germany) containing 1 mL ice cold phosphate-buffered saline and 5 mM butylated hydroxytoluene (antioxidant) by homogenizing 3 times at 600 rpm for 30 s in the MagNA Lyser Instrument (Roche Diagnostics, Mannheim, Germany). Supernatant was collected and stored at −80 °C for batch analysis. Superoxide dismutase (SOD) activity was determined using the commercially available SOD Colorimetric Activity Kit by Thermo Fisher Scientific (Waltham, MA, USA) exactly as described by the manufacturer. The BCA Protein Assay Kit (Thermo Fisher Scientific, Waltham, MA, USA) was used to adjust values to total protein levels. Results are expressed as units per mg of protein.

### 4.7. Blood Sample Analysis

Venous blood samples were collected from jugular veins before diet administration and at the end of the experiment at terminal blood collection from vena cava inferior under general anesthesia. Blood cells were separated from serum at 1970× *g* at 4 °C for 10 min and subsequently stored at −80 °C for further analyses. Determination of serum glycine levels was performed in the routine hospital laboratory.

### 4.8. Statistical Analysis

Statistical analyses were performed using SPSS (Statistical Package for the Social Sciences) version 23.0 (IBM Corp., Armonk, NY, USA). The Kruskal-Wallis test was used to compare sham and IRI groups (comparison of more than two groups with Bonferroni correction). Experimental groups with representative control groups (comparison of two groups) were analyzed using the Mann-Whitney U test. Data are presented as median and quartiles (Q1; Q3). A *p* value less than 0.05 was considered as statistically significant.

## 5. Conclusions

Pretreatment with melatonin and glycine provided protection against IRI in a rat warm ischemia model. This study represents a step toward understanding the effects of melatonin and glycine, which are commonly used as cytoprotective agents due to their antioxidative and anti-inflammatory properties in IRI. Since both are natural and nontoxic molecules, their use in UTx is considered safe. Although dietary melatonin and glycine, given separately, exert beneficial effects, the combined supplementation did not yield additive properties. Further investigations replicating the clinical situation are warranted in order to pave the way for clinical studies focusing on new organ-protective strategies in UTx using glycine, melatonin or their combination.

## Figures and Tables

**Table 1:** Results of the scoring system for uterus IRI evaluation.

	Sham Procedure				IRI Procedure			
	Control	Melatonin	Glycine	Melatonin + Glycine	Control	Melatonin	Glycine	Melatonin + Glycine
Inflammatory cells	1 (0.75; 1)	1 (0.75; 1)	1 (1; 1)	1 (0.5; 1)	2 (1; 2)	1 (1; 1.25)	1 (1; 2)	1 (1; 1.25)
Vasoconstriction	0 (0; 0.25)	0 (0; 0)	0 (0; 0.25)	0 (0; 0)	0 (0; 1)	0 (0; 1)	0 (0; 0.25)	0 (0; 0.25)
Hemorrhage	0 (0; 0)	0 (0; 0)	0 (0; 0)	0 (0; 0)	0 (0; 0)	0 (0; 0)	0 (0; 0)	0 (0; 0)
Necrosis	1 (1; 1)	1 (0.75; 1)	1 (0.75; 1)	1 (1; 1)	1 (1; 2)	1 (1; 1.25)	1 (1; 2)	1 (1; 1.25)
Edema	1 (0.75; 1)	1 (0; 1)	1 (1; 1)	1 (0; 1)	2 (1; 2)	1 (1; 2)	1 (1; 2)	1 (1; 1)
Thrombosis	0 (0; 0)	0 (0; 0)	0 (0; 0)	0 (0; 0)	0 (0; 0.25)	0 (0; 0)	0 (0; 0)	0 (0; 0)
Endometrial loss of cells	1 (1; 1)	1 (0.75; 1)	1 (0.75; 1)	1 (0.5; 1)	1 (1; 2)	1 (1; 1.25)	1 (1; 2)	1 (1; 1)
Smooth muscle contraction	0 (0; 0)	0 (0; 0)	0 (0; 0)	0 (0; 1)	0 (0; 1)	0 (0; 1)	1 (0; 1)	0 (0; 0.25)
Impaired basement membrane integrity	1 (0; 1)	0 (0; 1)	0 (0; 0.25)	0 (0; 1)	1 (0; 1)	0.5 (0; 1)	1 (0; 1)	1 (0.75; 1)
Perimeter thickening	0 (0; 0.25)	0 (0; 1)	0.5 (0; 1)	1 (0; 1)	1 (0.75; 1)	0.5 (0; 1)	0 (0; 1)	0.5 (0; 1)
Total score	5 (4; 6)	4 (3; 6)	4.5 (4; 6)	4 (3.5; 6)	8.5 (7; 10.25)	6 (5; 8)	7 (6; 8)	6 (6; 7)

IRI: ischemia/reperfusion injury. Data presented as median and quartiles (Q1; Q3); *n* = 10/group, except *n* = 9 in sham combined treatment group.

**Table 2:** Scoring system for the evaluation of uterus IRI.

	Score		
	0	1	2
Inflammatory cells	Absent	Moderate number of cells	Severe infiltration of cells
Vasoconstriction	Absent	<20% of small vessels	>20% of small vessels
Hemorrhage	Absent	Subendometrial	Myometrial and endometrial
Necrosis	Absent	<20%	>20%
Edema	Absent	<50%	>50%
Thrombosis	Absent	<50% of the vessels	>50% of the vessels
Endometrial loss of cells	Absent	<20%	>20%
Smooth muscle contraction	Absent	Present	
Impaired basement membrane integrity	Absent	Present	
Perimeter thickening	Absent	Present	

Maximum score for uterus IRI—17. IRI: ischemia/reperfusion injury.

## Data Availability

The data that support the findings of this study are available from the first author (V.Z.) upon reasonable request.

## Supplementary Materials

The following are available online at https://www.mdpi.com/article/10.3390/ijms22168373/s1.

## References

[1] Brannstrom M., Enskog A., Kvarnstrom N., Ayoubi J.M., Dahm-Kahler P. (2019). Global results of human uterus transplantation and strategies for pre-transplantation screening of donors. Fertil. Steril..

[2] Jones B.P., Saso S., Bracewell-Milnes T., Thum M.Y., Nicopoullos J., Diaz-Garcia C., Friend P., Ghaem-Maghami S., Testa G., Johannesson L. (2019). Human uterine transplantation: A review of outcomes from the first 45 cases. BJOG.

[3] Ozkan O., Dogan N.U., Ozkan O., Mendilcioglu I., Dogan S., Aydinuraz B., Simsek M. (2016). Uterus transplantation: From animal models through the first heart beating pregnancy to the first human live birth. Women’s Health.

[4] Zitkute V., Kvietkauskas M., Leber B., Strupas K., Stiegler P., Schemmer P. (2020). Ischemia and reperfusion injury in uterus transplantation: A comprehensive review. Transplant. Rev..

[5] O’Donovan L., Williams N.J., Wilkinson S. (2019). Ethical and policy issues raised by uterus transplants. Br. Med. Bull..

[6] Brannstrom M., Dahm-Kahler P. (2019). Uterus transplantation and fertility preservation. Best Pract. Res. Clin. Obstet. Gynaecol..

[7] Castellon L.A.R., Amador M.I.G., Gonzalez R.E.D., Eduardo M.S.J., Diaz-Garcia C., Kvarnstrom N., Branstrom M. (2017). The history behind successful uterine transplantation in humans. JBRA Assist. Reprod..

[8] Halladin N.L. (2015). Oxidative and inflammatory biomarkers of ischemia and reperfusion injuries. Dan. Med. J..

[9] Wu M.Y., Yiang G.T., Liao W.T., Tsai A.P., Cheng Y.L., Cheng P.W., Li C.Y., Li C.J. (2018). Current Mechanistic Concepts in Ischemia and Reperfusion Injury. Cell. Physiol. Biochem..

[10] Eltzschig H.K., Eckle T. (2011). Ischemia and reperfusion--from mechanism to translation. Nat. Med..

[11] Horvat M., Iltis A. (2019). What Are Good Guidelines for Evaluating Uterus Transplantation?. AMA J. Ethics.

[12] Stiegler P., Bausys A., Leber B., Strupas K., Schemmer P. (2018). Impact of Melatonin in Solid Organ Transplantation—Is It Time for Clinical Trials? A Comprehensive Review. Int. J. Mol. Sci..

[13] Zitkute V., Kvietkauskas M., Maskoliunaite V., Leber B., Ramasauskaite D., Strupas K., Stiegler P., Schemmer P. (2020). Custodiol-N Is Superior to Custodiol((R)) Solution in Experimental Rat Uterus Preservation. Int. J. Mol. Sci..

[14] Slominski R.M., Reiter R.J., Schlabritz-Loutsevitch N., Ostrom R.S., Slominski A.T. (2012). Melatonin membrane receptors in peripheral tissues: Distribution and functions. Mol. Cell. Endocrinol..

[15] Crupi R., Mazzon E., Marino A., La Spada G., Bramanti P., Spina E., Cuzzocrea S. (2011). Melatonin’s stimulatory effect on adult hippocampal neurogenesis in mice persists after ovariectomy. J. Pineal Res..

[16] Esteban-Zubero E., Garcia-Gil F.A., Lopez-Pingarron L., Alatorre-Jimenez M.A., Inigo-Gil P., Tan D.X., Garcia J.J., Reiter R.J. (2016). Potential benefits of melatonin in organ transplantation: A review. J. Endocrinol..

[17] Campolo M., Ahmad A., Crupi R., Impellizzeri D., Morabito R., Esposito E., Cuzzocrea S. (2013). Combination therapy with melatonin and dexamethasone in a mouse model of traumatic brain injury. J. Endocrinol..

[18] Fusco R., Siracusa R., D’Amico R., Peritore A.F., Cordaro M., Gugliandolo E., Crupi R., Impellizzeri D., Cuzzocrea S., Di Paola R. (2019). Melatonin Plus Folic Acid Treatment Ameliorates Reserpine-Induced Fibromyalgia: An Evaluation of Pain, Oxidative Stress, and Inflammation. Antioxidants.

[19] Olcese J.M. (2020). Melatonin and Female Reproduction: An Expanding Universe. Front. Endocrinol..

[20] Saat N., Risvanli A., Dogan H., Onalan E., Akpolat N., Seker I., Sahna E. (2019). Effect of melatonin on torsion and reperfusion induced pathogenesis of rat uterus. Biotech. Histochem..

[21] Turkoz Y., Celik O., Hascalik S., Cigremis Y., Hascalik M., Mizrak B., Yologlu S. (2004). Melatonin reduces torsion-detorsion injury in rat ovary: Biochemical and histopathologic evaluation. J. Pineal Res..

[22] Aslaner A., Gunal O., Turgut H.T., Celik E., Yildirim U., Demirci R.K., Gunduz U.R., Calis H., Dogan S. (2013). Effect of melatonin on kidney cold ischemic preservation injury. Int. J. Clin. Exp. Med..

[23] Petrat F., Boengler K., Schulz R., de Groot H. (2012). Glycine, a simple physiological compound protecting by yet puzzling mechanism(s) against ischaemia-reperfusion injury: Current knowledge. Br. J. Pharmacol..

[24] Habib M.M., Hodgson H.J., Davidson B.R. (2006). The role of glycine in hepatic ischemia-reperfusion injury. Curr. Pharm. Des..

[25] Petrat F., Drowatzky J., Boengler K., Finckh B., Schmitz K.J., Schulz R., de Groot H. (2011). Protection from glycine at low doses in ischemia-reperfusion injury of the rat small intestine. Eur. Surg. Res..

[26] Zhong X., Li X., Qian L., Xu Y., Lu Y., Zhang J., Li N., Zhu X., Ben J., Yang Q. (2012). Glycine attenuates myocardial ischemia-reperfusion injury by inhibiting myocardial apoptosis in rats. J. Biomed. Res..

[27] Maneikyte J., Bausys A., Leber B., Feldbacher N., Hoefler G., Kolb-Lenz D., Strupas K., Stiegler P., Schemmer P. (2020). Dietary Glycine Prevents FOLFOX Chemotherapy-Induced Heart Injury: A Colorectal Cancer Liver Metastasis Treatment Model in Rats. Nutrients.

[28] Maneikyte J., Bausys A., Leber B., Horvath A., Feldbacher N., Hoefler G., Strupas K., Stiegler P., Schemmer P. (2019). Dietary glycine decreases both tumor volume and vascularization in a combined colorectal liver metastasis and chemotherapy model. Int. J. Biol. Sci..

[29] Brannstrom M., Wranning C.A., Altchek A. (2010). Experimental uterus transplantation. Hum. Reprod. Update.

[30] Haddad C.F., Haddad J.M., Veiga E.C.A., Sorpreso I.C.E., Simoes R.S., Baracat E.C., Soares Junior J.M. (2020). Melatonin and organ transplantation: What is the relationship?. Rev. Assoc. Med. Bras. (1992).

[31] Jung F.J., Yang L., Harter L., Inci I., Schneiter D., Lardinois D., Keel M., Weder W., Korom S. (2004). Melatonin in vivo prolongs cardiac allograft survival in rats. J. Pineal Res..

[32] Liu C., Hong T., Shao M., Chen Z., Wang C. (2014). Melatonin synergized with cyclosporine A improves cardiac allograft survival by suppressing inflammation and apoptosis. Mol. Med. Rep..

[33] Zhou H., Ma Q., Zhu P., Ren J., Reiter R.J., Chen Y. (2018). Protective role of melatonin in cardiac ischemia-reperfusion injury: From pathogenesis to targeted therapy. J. Pineal Res..

[34] Takechi M., Tatehara S., Satomura K., Fujisawa K., Nagayama M. (2008). Effect of FGF-2 and melatonin on implant bone healing: A histomorphometric study. J. Mater. Sci. Mater. Med..

[35] Inci I., Inci D., Dutly A., Boehler A., Weder W. (2002). Melatonin Attenuates Posttransplant Lung Ischemia-Reperfusion Injury. Ann. Thorac. Surg..

[36] Lin G.J., Huang S.H., Chen Y.W., Hueng D.Y., Chien M.W., Chia W.T., Chang D.M., Sytwu H.K. (2009). Melatonin prolongs islet graft survival in diabetic NOD mice. J. Pineal Res..

[37] Munoz-Casares F.C., Padillo F.J., Briceno J., Collado J.A., Munoz-Castaneda J.R., Ortega R., Cruz A., Tunez I., Montilla P., Pera C. (2006). Melatonin reduces apoptosis and necrosis induced by ischemia/reperfusion injury of the pancreas. J. Pineal Res..

[38] Panah F., Ghorbanihaghjo A., Argani H., Haiaty S., Rashtchizadeh N., Hosseini L., Dastmalchi S., Rezaeian R., Alirezaei A., Jabarpour M. (2019). The effect of oral melatonin on renal ischemia-reperfusion injury in transplant patients: A double-blind, randomized controlled trial. Transpl. Immunol..

[39] Shi S., Lei S., Tang C., Wang K., Xia Z. (2019). Melatonin attenuates acute kidney ischemia/reperfusion injury in diabetic rats by activation of the SIRT1/Nrf2/HO-1 signaling pathway. Biosci. Rep..

[40] Li Z., Nickkholgh A., Yi X., Bruns H., Gross M.L., Hoffmann K., Mohr E., Zorn M., Buchler M.W., Schemmer P. (2009). Melatonin protects kidney grafts from ischemia/reperfusion injury through inhibition of NF-kB and apoptosis after experimental kidney transplantation. J. Pineal Res..

[41] Esteban-Zubero E., Garcia-Gil F.A., Lopez-Pingarron L., Alatorre-Jimenez M.A., Ramirez J.M., Tan D.X., Garcia J.J., Reiter R.J. (2016). Melatonin role preventing steatohepatitis and improving liver transplantation results. Cell. Mol. Life Sci..

[42] Mortezaee K., Khanlarkhani N. (2018). Melatonin application in targeting oxidative-induced liver injuries: A review. J. Cell. Physiol..

[43] Kim S.H., Lee S.M. (2008). Cytoprotective effects of melatonin against necrosis and apoptosis induced by ischemia/reperfusion injury in rat liver. J. Pineal Res..

[44] Liang R., Nickkholgh A., Hoffmann K., Kern M., Schneider H., Sobirey M., Zorn M., Buchler M.W., Schemmer P. (2009). Melatonin protects from hepatic reperfusion injury through inhibition of IKK and JNK pathways and modification of cell proliferation. J. Pineal Res..

[45] Koh P.O. (2011). Melatonin prevents hepatic injury-induced decrease in Akt downstream targets phosphorylations. J. Pineal Res..

[46] Hadj Ayed Tka K., Mahfoudh Boussaid A., Zaouali M.A., Kammoun R., Bejaoui M., Ghoul Mazgar S., Rosello Catafau J., Ben Abdennebi H. (2015). Melatonin modulates endoplasmic reticulum stress and Akt/GSK3-beta signaling pathway in a rat model of renal warm ischemia reperfusion. Anal. Cell. Pathol..

[47] Song J., Kang S.M., Lee W.T., Park K.A., Lee K.M., Lee J.E. (2014). The beneficial effect of melatonin in brain endothelial cells against oxygen-glucose deprivation followed by reperfusion-induced injury. Oxid. Med. Cell. Longev..

[48] Schemmer P., Bradford B.U., Rose M.L., Bunzendahl H., Raleigh J.A., Lemasters J.J., Thurman R.G. (1999). Intravenous glycine improves survival in rat liver transplantation. Am. J. Physiol..

[49] Wheeler M.D., Ikejema K., Enomoto N., Stacklewitz R.F., Seabra V., Zhong Z., Yin M., Schemmer P., Rose M.L., Rusyn I. (1999). Glycine: A new anti-inflammatory immunonutrient. Cell. Mol. Life Sci. CMLS.

[50] Wang W., Wu Z., Lin G., Hu S., Wang B., Dai Z., Wu G. (2014). Glycine stimulates protein synthesis and inhibits oxidative stress in pig small intestinal epithelial cells. J. Nutr..

[51] Lu Y., Zhang J., Ma B., Li K., Li X., Bai H., Yang Q., Zhu X., Ben J., Chen Q. (2012). Glycine attenuates cerebral ischemia/reperfusion injury by inhibiting neuronal apoptosis in mice. Neurochem. Int..

[52] Kvietkauskas M., Leber B., Strupas K., Stiegler P., Schemmer P. (2020). Machine Perfusion of Extended Criteria Donor Organs: Immunological Aspects. Front. Immunol..

[53] Kvietkauskas M., Zitkute V., Leber B., Strupas K., Stiegler P., Schemmer P. (2021). Dietary Melatonin and Glycine Decrease Tumor Growth through Antiangiogenic Activity in Experimental Colorectal Liver Metastasis. Nutrients.

[54] Mikalauskas S., Mikalauskiene L., Bruns H., Nickkholgh A., Hoffmann K., Longerich T., Strupas K., Buchler M.W., Schemmer P. (2011). Dietary glycine protects from chemotherapy-induced hepatotoxicity. Amino Acids.

[55] Johannesson L., Enskog A., Dahm-Kahler P., Hanafy A., Chai D.C., Mwenda J.M., Diaz-Garcia C., Olausson M., Brannstrom M. (2012). Uterus transplantation in a non-human primate: Long-term follow-up after autologous transplantation. Hum. Reprod..

[56] Sahin S., Ozakpinar O.B., Ak K., Eroglu M., Acikel M., Tetik S., Uras F., Cetinel S. (2014). The protective effects of tacrolimus on rat uteri exposed to ischemia-reperfusion injury: A biochemical and histopathologic evaluation. Fertil. Steril..

[57] Atalay Y.O., Aktas S., Sahin S., Kucukodaci Z., Ozakpinar O.B. (2015). Remifentanil protects uterus against ischemia-reperfusion injury in rats. Acta Cir. Bras..

[58] Siracusa R., Impellizzeri D., Cordaro M., Gugliandolo E., Peritore A.F., Di Paola R., Cuzzocrea S. (2018). Topical Application of Adelmidrol + Trans-Traumatic Acid Enhances Skin Wound Healing in a Streptozotocin-Induced Diabetic Mouse Model. Front. Pharmacol..

[59] Diaz-Garcia C., Akhi S.N., Martinez-Varea A., Brannstrom M. (2013). The effect of warm ischemia at uterus transplantation in a rat model. Acta Obstet. Gynecol. Scand..

[60] Sahin Ersoy G., Kurek Eken M., Cevik O., Cilingir O.T., Tal R. (2017). Mycophenolate mofetil attenuates uterine ischaemia/reperfusion injury in a rat model. Reprod. Biomed. Online.

[61] Fusco R., Gugliandolo E., Siracusa R., Scuto M., Cordaro M., D’Amico R., Evangelista M., Peli A., Peritore A.F., Impellizzeri D. (2020). Formyl Peptide Receptor 1 Signaling in Acute Inflammation and Neural Differentiation Induced by Traumatic Brain Injury. Biology.

[62] Bankhead P., Loughrey M.B., Fernandez J.A., Dombrowski Y., McArt D.G., Dunne P.D., McQuaid S., Gray R.T., Murray L.J., Coleman H.G. (2017). QuPath: Open source software for digital pathology image analysis. Sci. Rep..

