# Supplementary material for: Melatonin and Glycine Reduce Uterus Ischemia/Reperfusion Injury in a Rat Model of Warm Ischemia
Source: Int J Mol Sci. 2021 Aug 4;22(16):8373. doi: 10.3390/ijms22168373 (PMC8394613; doi:10.3390/ijms22168373)
Supplement: Supplementary file 1 [file ijms-22-08373-s001.zip › Supplementary Figure S1.pdf]

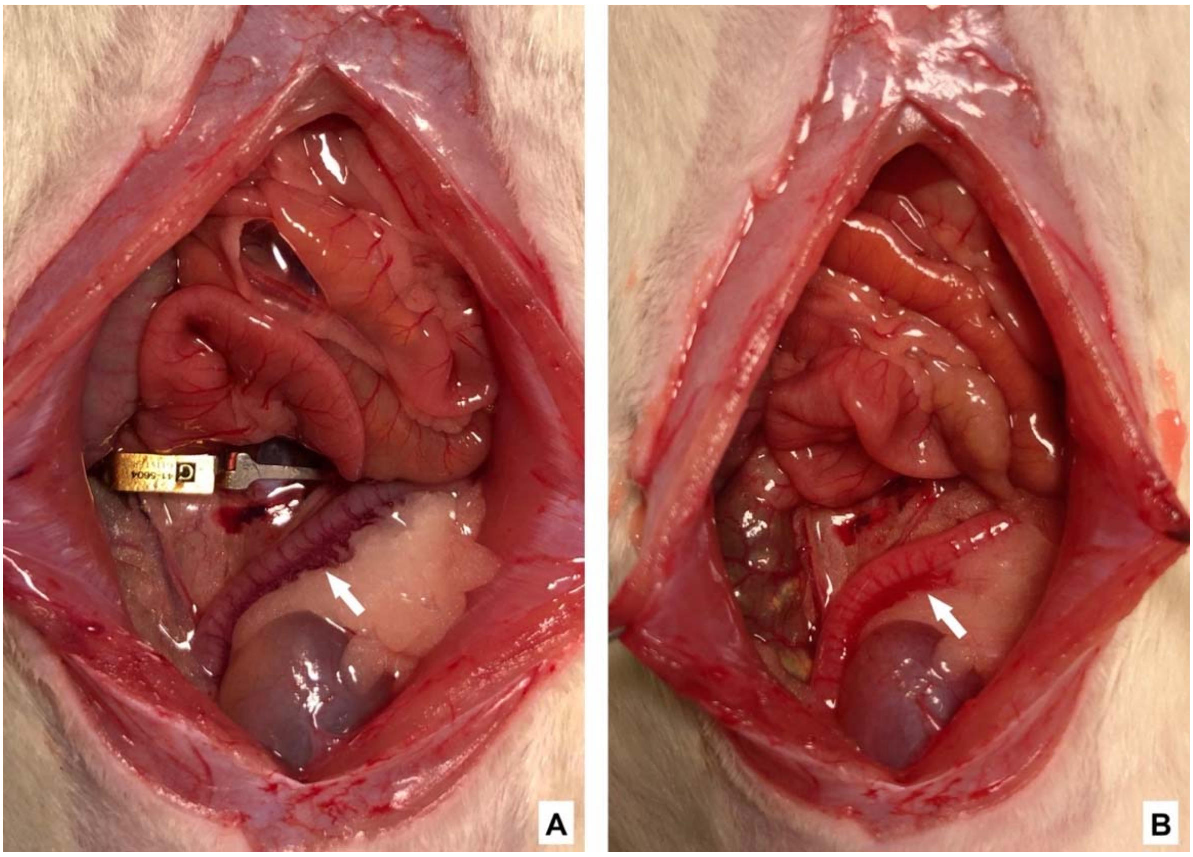

Figure S1. Visual changes in uterus during ischemia and reperfusion. A; uterus (white arrow) after 1 h of ischemia. B; uterus (white arrow) after 1 h of ischemia followed by 1 h of reperfusion.
