# Supplementary material for: Melatonin and Glycine Reduce Uterus Ischemia/Reperfusion Injury in a Rat Model of Warm Ischemia
Source: Int J Mol Sci. 2021 Aug 4;22(16):8373. doi: 10.3390/ijms22168373 (PMC8394613; doi:10.3390/ijms22168373)
Supplement: Supplementary file 1 [file ijms-22-08373-s001.zip › Supplementary Information.pdf]

Supplementary Information

Criteria for evaluation of uterus IRI scoring system

The presence of scattered polymorphonuclear cells in the tissue was considered as moderate inflammation (score 1), formation of small clusters of these cells was evaluated as severe inflammation (score 2). Vasoconstriction was identified by the contraction of the endothelial lining in small vessels. In addition, vessels with thrombi in the lumen were counted. Hemorrhages were identified in different layers of the uterus: subendometrial (score 1) or myometrial and endometrial (score 2). Percent of necrosis, edema, or endometrial loss of cells, was given according to the area of damage and whole tissue. Smooth muscle contraction was present when muscle fibers had a tortuous appearance in part or full thickness of the myometrium (score 1). The integrity of the basement membrane was considered as impaired (score 1) when subendothelial eosinophilic material was thinned and had small or larger fractions (score 1). Perimeter thickening was present in slides with a thicker, slightly wavy layer of connective tissue in the outside of the uterus (score 1).
